# First publication of endemic channels as part of a pediatric Antimicrobial Stewardship Program: when to turn on the alarms? Recommendations of a pediatric ASP program

**DOI:** 10.1186/s12879-022-07916-z

**Published:** 2023-01-11

**Authors:** Juan Pablo Londoño-Ruiz, Ivan Felipe Gutierrez-Tobar, Naddya Lheidy Bermúdez-Bohórquez, Andrea Esperanza Rodríguez

**Affiliations:** 1 Antimicrobial Stewardship Program, Clinica Infantil Colsubsidio, Bogotá, Colombia; 2 Pediatric Infectious Diseases, Clinica Infantil Santa Maria del Lago, Bogotá, Colombia; 3 Pharmacy Department, Clinica Infantil Colsubsidio, Bogotá, Colombia; 4 Colsubsidio Investiga, Clinica Infantil Colsubsidio, Bogotá, Colombia; 5 Department of Pediatrics, Universidad del Rosario, Bogota, Colombia

**Keywords:** Antimicrobial Stewardship, Rational antibiotic use, Pediatrics

## Abstract

**Background:**

Pediatric Antimicrobial Stewardship Programs (ASP) consider DOT a fundamental measure to quantify the impact of ASP. Novel strategies have been described, but no endemic channels (EC) have been reported to compare antibiotic use within historical patterns. This report describes the process of constructing an EC and analyzing its interpretation.

**Methods:**

This was a descriptive study of the construction, implementation, and analysis of EC. The median and quartile method, as well as the geometric mean (GM) and confidence interval (CI) methods using DOT for the last 4 years were used. ECs have also been elaborated on in critical services (PICU).

**Results:**

GM and CI method seem to be more sensitive in identifying changes in antimicrobial use. Ceftriaxone increased its use starting in December 2021, reaching the warning zone in March 2022 in relation to increased cases of bacterial and complicated pneumonia. Piperacillin–tazobactam showed an important increase in PICU during the first 8 months of 2021, reaching the alert zone until August 2021; thereafter, its use decreased, and this variation was related to a modification in the presentation of complicated appendicitis during the COVID 19 pandemic restrictions. The use of ampicillin-sulbactam has increased since January 2022 because of a change in local guidelines regarding its use in appendicitis and peritonitis. The changes identified in each EC allowed ASP to take different conducts.

**Conclusion:**

EC allowed us to construct a new tool to measure ASP impact, internal comparison of antibiotic use facilitated taking timely interventions. EC could be useful for all pediatric and adult ASP.

## Background

Antimicrobial Stewardship Programs (ASP) are the standard of care for the use of antibiotics in pediatric hospitals. The quantification of the impact on ASP is a core element of every program [1, 2]. Different strategies have been proposed to measure the impact of ASP, including resistance, costs, *Clostridium difficile* infections, and length of hospitalization [1–4]. Measurement of antimicrobial consumption is the most commonly used method and focuses on daily doses (DDD) and days of therapy (DOT) [5].

The World Health Organization and pediatric ASP recommendations consider the DOT of antibiotics as a fundamental measure in pediatric hospitals to quantify the impact of ASPs [3, 4, 6]. DOT is defined as the number of days a patient receives an antibiotic independent of dose, in which the numerator represents days of therapy with an agent during a period, and the denominator is the total number of patient-days within that period multiplied by 1000, to obtain data per 1000 patient-days [5, 7].

Endemic channels are a graphic representation of surveillance data that allows for interpretation in the context of historical data, whether new cases of a disease, or antibiotic consumption. Various methods are described in the literature, but all of them consist of calculating a measure of central tendency and a typical fluctuation path of the incidence for each month. This methodology has been used in the study of infectious diseases that show a seasonal behavior to define when there is a significant increase (or decrease) in the number of cases compared to historical cases, as is the case of Dengue in Colombia [8].

Novel strategies to quantify ASP impact have been described, including adjustment of antimicrobial use by quality of care and institutional microbiological burden [9], as well as the standardized antimicrobial administration ratio, a metric for measuring and comparing antibiotic use [10]. According to our literature search and experience, no study has reported the use and analysis of endemic channels (EC) to compare antibiotic use within historical patterns. This report describes the process of constructing endemic channels to evaluate antimicrobial consumption as part of a pediatric ASP and analyses its interpretation more than 1 year after its implementation.

## Methods

A descriptive study was conducted with a focus on antimicrobial surveillance, describing the construction, implementation, and analysis of endemic channels to monitor antimicrobial use at a third-level reference pediatric center in Bogota, Colombia, South America, at Clínica Infantil Colsubsidio, a reference center with 185 beds and most pediatric specialties.

Antimicrobial Stewardship Program methodology: We have had the ASP at our center since 2016; initially (2016–2019), its main focus was on broad-spectrum antibiotics, and by 2019, narrow-spectrum antibiotics were included. The ASP is co-led by an interdisciplinary team consisting of two ID physicians, a pharmacist, a microbiology team, epidemiology support, different medical department leaders and important administrative support. The main focus of our ASP is persuasive strategies, such as educational methodologies, audits and feedback, guidelines, and local algorithm implementation.

**Fig. 1:**
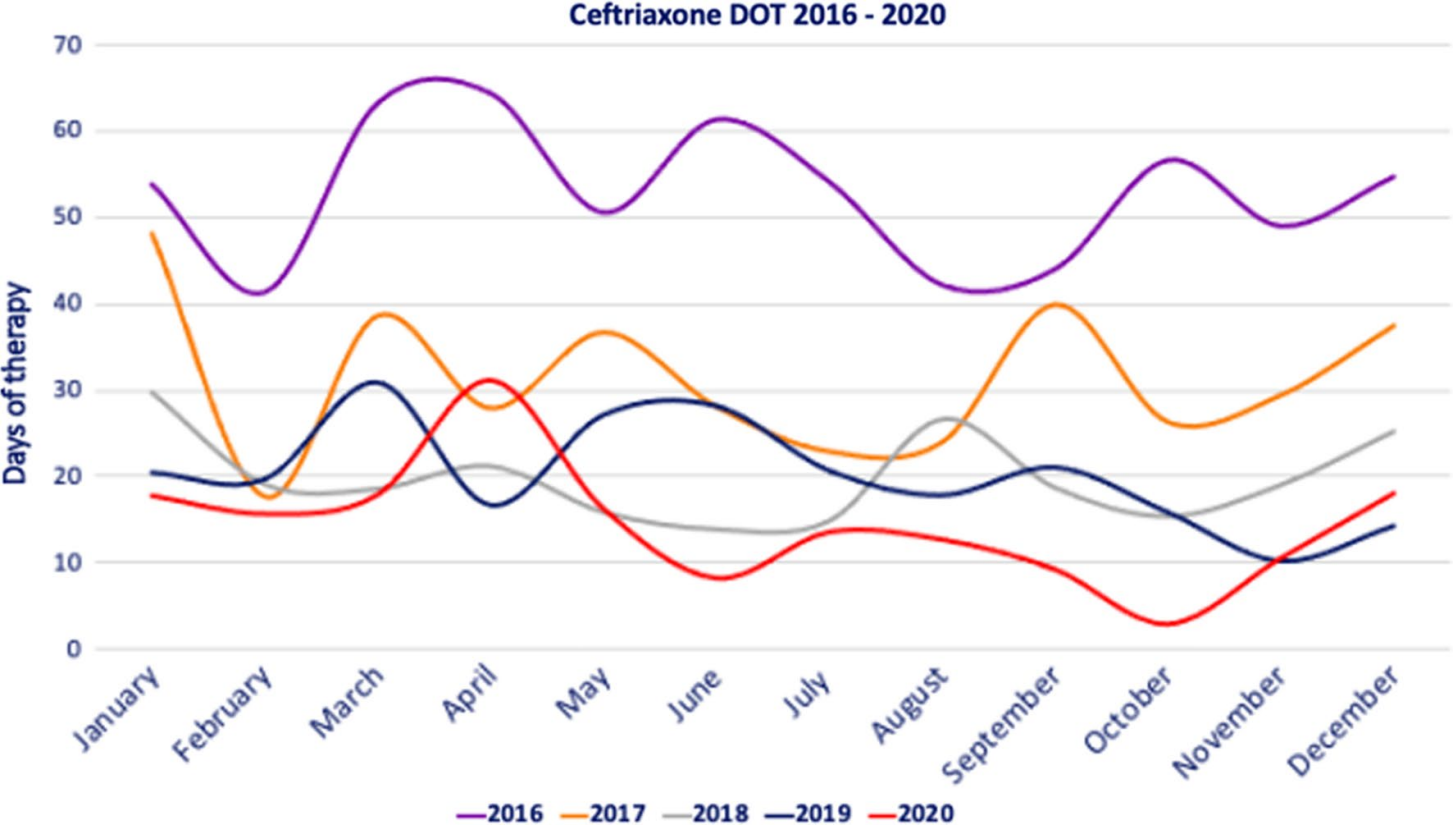
Ceftriaxone DOT 2016–2020. This methodology allows the construction of endemic channels for each of the monitored antibiotics. Although our ASP reports the endemic channels individually (for the monitored antibiotics), it would be possible to present them grouped by type of antibiotic, for example, carbapenems, or penicillins

Endemic channel elaboration: We have measured DOT since 2016, and as part of endemic channel construction, we included DOT for the last 4 years (2017–2020) from all included antibiotics per year and month. We intentionally excluded DOT for 2016 because this was the year the ASP began and consumption was higher than in subsequent years. Since 2017, antibiotic consumption has been closer to current consumption (Fig. 1).

We used the median and quartile method, as well as the geometric mean (GM) and confidence interval (CI) method. Whenever DOT was zero (non-antibiotic consumed during that month), we adjusted the formula by adding one (+ 1) to this value (included in endemic channel construction and the actual DOT) to avoid the problem of an indeterminate value of the natural logarithm. We use the Bortman method [11] to construct endemic channels. In general, a measure of central tendency (median or geometric mean, depending on the method used) should be calculated, and then an upper and lower channel (which can be upper and lower quartiles or confidence intervals) should be elaborated.

With both methods, four zones were generated: one below the lower limit or success zone (< p25 or inferior limit of CI, depending on the method used), one between the lower limit and median or geometric mean (p25–p50 or between the inferior limit of CI and geometric mean) or safety zone, one between the median and upper limit or alert zone (p50–p75 or between geometric mean and superior limit of CI), and one above the upper limit (> p75 or superior limit of CI) or epidemic zone [11].

In addition to constructing an EC to evaluate general consumption at the hospital, we also constructed a specific EC for critical services (Pediatric Intensive Care Unit and Oncology Service).

## Results

Construction and implementation: Using both methods, medians, and quartiles, as well as geometric means and confidence intervals [11], we elaborated ceftriaxone EC, and general calculations are presented in Table 1.

**Table 1:** DOT* of ceftriaxone (2017–2020) and calculated quartiles

Month	Days of therapy (DOT) Ceftriaxone				Quartile method**			Geometric mean (GM) and confidence intervals (CI) method^		
	2017	2018	2019	2020	Q1	Q2	Q3	Superior CI	GM	Inferior CI
January	48	30	20	18	18	20	30	3.75	3.09	2.43
February	18	19	20	16	16	19	20	3.20	2.89	2.58
March	39	19	31	18	18	19	31	3.83	3.08	2.32
April	28	21	17	31	17	21	31	3.89	3.10	2.32
May	37	16	27	16	16	16	27	3.70	2.95	2.21
June	28	14	28	8	8	14	28	4.23	2.69	1.15
July	23	15	21	14	14	15	21	3.33	2.78	2.23
August	24	27	18	13	13	18	27	3.82	2.90	1.98
September	40	19	21	9	9	19	21	3.85	2.73	1.61
October	26	15	16	3	3	15	16	4.64	2.18	0.00
November	29	19	10	10	10	10	19	3.42	2.54	1.66
December	37	25	14	18	14	18	25	3.64	2.92	2.21

*DOT × 1000 patient-days

^**^Q1: percentile 25, Q2: percentile 50, Q3: percentile 75

^DOT (days of therapy) used to construct endemic channel and depicted in GM and CI methods should be in natural logarithm

**Fig. 2:**
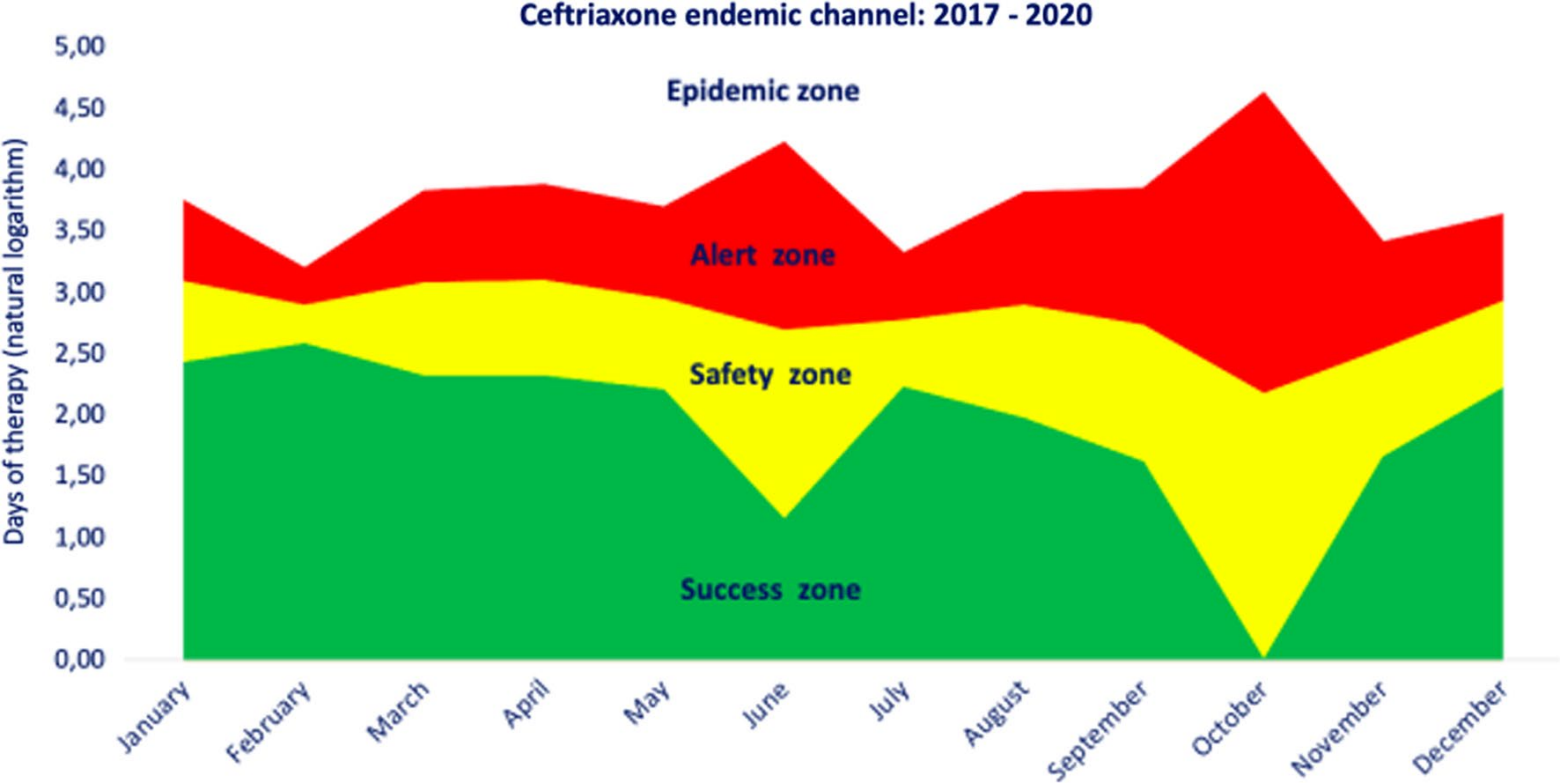
Ceftriaxone endemic channel: 2017–2020

As mentioned, different zones of antimicrobial use were defined according to historic DOTs. Figure 2 describes the variation in the historical use of ceftriaxone, with a slight increment in the last 3 months of the year and again from February to March, in concordance with the most intense respiratory infections and rainy season in Bogotá, which is associated with increased use of ceftriaxone because of more frequent cases of bacterial pneumonia and complicated pneumonia. In this historical use, the actual use was compared.

According to each zone, ASP analysis EC as follows: a value located in the success zone indicates that the use of antibiotics (DOT) for that period has a lower frequency than expected, one located in the security zone indicates that the antibiotic consumption shows a stable behavior, and a value located in the alert zone indicates that the number has a higher frequency than expected, and it is necessary to study the situation to determine the origin of the variation. A value located in the epidemic zone indicates that the DOTs reported for that period present a situation that requires immediate control actions.

**Fig. 3:**
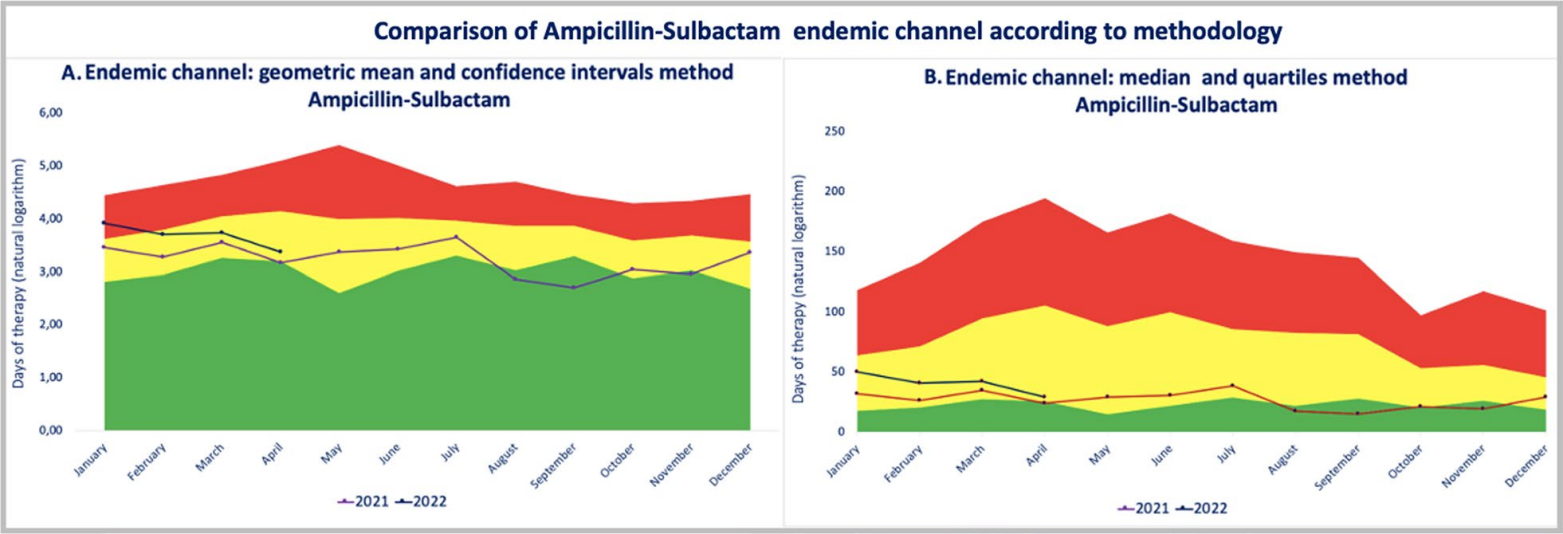
Comparison of Ampicillin-Sulbactam endemic channel according to methodology

Medians and quartiles Vs. geometric means and confidence interval method: When comparing both methods for the elaboration of endemic channels, we found that although the method of geometric means and confidence intervals requires much more data processing, making it more cumbersome, it is more sensitive than the median and quartiles method to detect changes in DOT with respect to the previous history of antimicrobial consumption, as can be observed in ampicillin sulbactam (AS) when a change in the guidelines of prophylaxis was made (Fig. 3).

Since January 2022, ampicillin-sulbactam has been the first indication as part of the primary surgical prophylaxis regimen for appendicitis. This change represents an increase in AS consumption. Using the median and quartile method (3B), this change appears only as a slight increase, but in the same zone (safety zone), while with the geometric mean and CI method (3A), the same change was more easily seen (change reached the alert zone). The possibility of detecting small differences in antimicrobial consumption, such as ASP, makes this model an ideal method for us (Fig. 3). For this reason, since 2020, in our institution, we decided to implement EC with geometric measurement of rates and confidence intervals using the natural logarithm for each DOT per month per year, mean, standard deviation, and Student’s t, to obtain upper and lower confidence intervals, which, in addition to being more sensitive according to our findings, have also been described in the literature as a more robust statistical data analysis method [11].

**Fig. 4:**
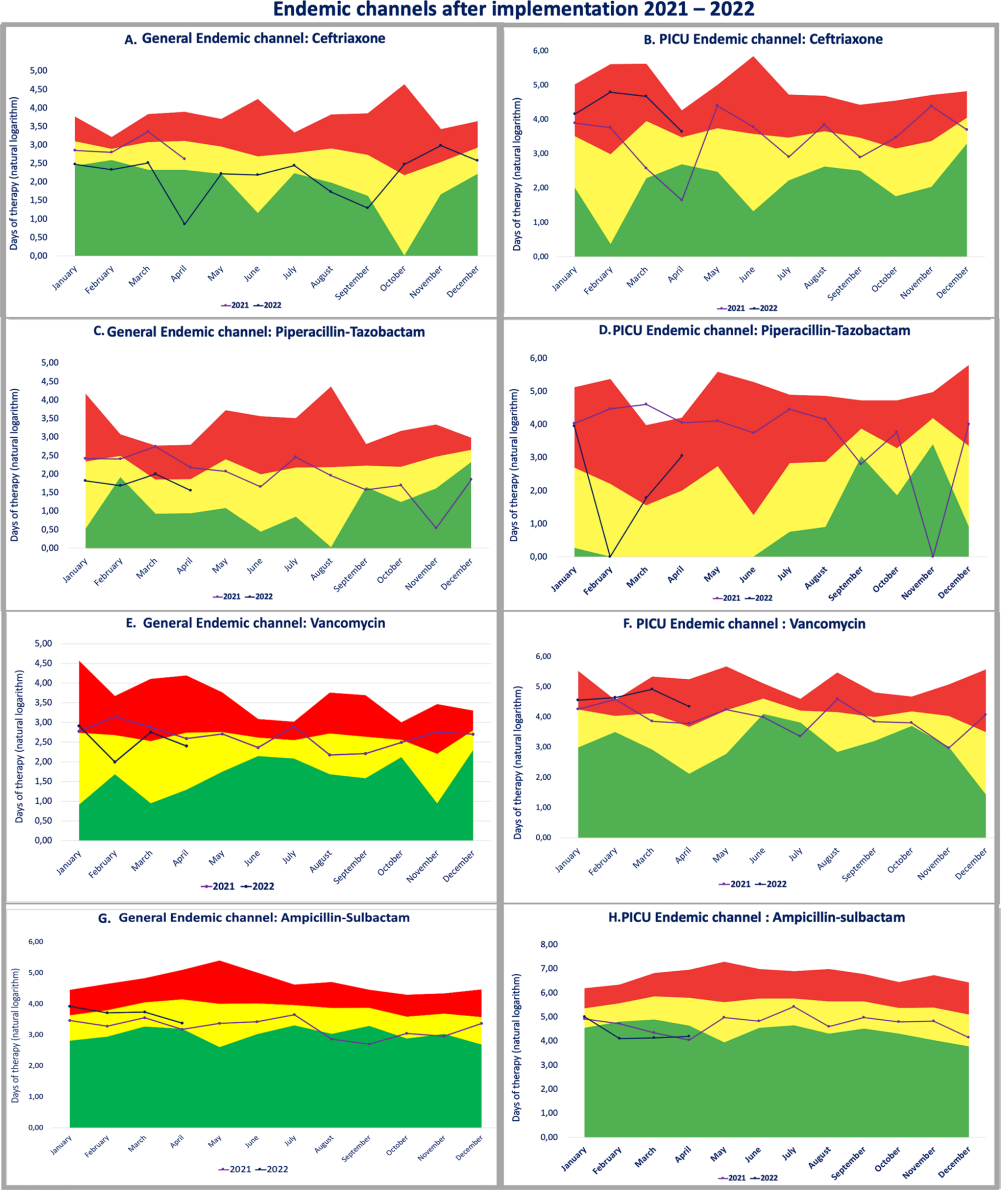
Endemic channels after implementation 2021–2022

One year after implementation: From 2021, every month, DOTs were analyzed in constructed endemic channels. In Fig. 4, we appreciate the behavior of three broad-spectrum antibiotics and a narrow-spectrum antibiotic (ampicillin-sulbactam) comparing ECs from all hospital services and the Pediatric Intensive Care Unit (PICU).

As shown in Fig. 4A, B, ceftriaxone presented an increase in consumption during the final trimester of 2021 and 2022, compared with previous years. While most of the time in 2021, its use was in the success zone (less than expected compared with previous years) in general hospital services, its use was more pronounced in the PICU, where it was in the alert zone most of the time.

In Fig. 4C, D Piperacillin–tazobactam (PT) showed an important increase in use with respect to historical consumption, especially in the PICU during the first 8 months of 2021, where its use was in the alert zone until August 2021. From September 2021, there is a decrease in PICU and general hospital use. During November and December 2021, its use was lower than that in the same period in previous years.

Vancomycin (Fig. 4E, F) has a relatively stable behavior; in general, it remains at the upper limit of the safety zone, but its use in the PICU has been greater than its historical use, especially during the first trimester of 2022. Ampicillin–sulbactam (Fig. 4G, H) has reported an increase in its use since January 2022, reaching the alert zone during this period. In the PICU, its use has been stable compared with historic consumption, remaining in the success zone during the first three months of the year in 2022, similar to what happened in the first trimester of 2021.

## Discussion

This strategy allowed us to construct a new tool to measure the impact of a pediatric antimicrobial stewardship program, facilitating timely interventions and comparisons with historical antimicrobial usage. After analyzing the two methods of EC construction, we implemented GM and IC methods [11] because of the increased sensitivity to detect consumption variations. Different channels allowed us to establish a measure of central tendency and the path of the normal fluctuation of specific antimicrobial use during the year. At present, this kind of strategy has not been recommended as part of the ASP measurement tool by any policy statement or ASP guidelines. The use of confidence intervals in prospective measurements is not possible since there is only one monthly value.

*Ceftriaxone* Concerning ceftriaxone, we could appreciate (Fig. 4A, B) an increase in its use compared with previous periods and years starting in December 2021, but especially in March 2022, when it almost reached the warning zone. During this period, at the hospital and as part of the evolution of the COVID-19 pandemic, children of all ages were reincorporated into the usual scholarly and social activities. At our hospital and in Bogota [12] similar to what has been described in other publications “new” behavior increased progressively emergency consults and hospitalizations related to respiratory diseases, including pneumonia, especially those caused by syncytial respiratory virus, rhinovirus, metapneumovirus, and a lesser degree SARS-CoV-2 [13]. Although penicillin was the first-line therapy in most cases when co-infection was suspected, some cases, especially during January and February, were associated with pneumococcal co-infection, complicated pneumonia, and some of them by resistant strains, as previously reported in Bogota [14]. As part of the ASP analysis and considering the endemic channel of ceftriaxone, we believe that part of this increase in ceftriaxone use was related to the increased severity of some identified cases, which led other clinicians to initiate ceftriaxone earlier. For this reason, from February to March, different educational strategies were implemented, and more intense ceftriaxone surveillance was performed. Although in April, we were still in a respiratory emergency, ceftriaxone use had declined in the last weeks. In this scenario, endemic channels allowed us to detect, analyze, and implement timely interventions to decrease the use of ceftriaxone.

*Piperacillin–tazobactam (PT)* as reported in some latitudes, COVID-19 pandemic, related restrictions, and fear of parents to assist hospitals, contributed to increases in complicated appendicitis, including peritonitis, appendicular plastron, and septic shock of abdominal origin, [15–17] PT represents an alternative, especially in severe cases of intra-abdominal surgical complications, and this in part was the conclusion of our analyses because no other modification besides severity of patients was found. In August 2021, all COVID-19 restrictions were precluded in Colombia, and fewer cases of complicated appendicitis have been reported since then, possibly related to a more timely consultation in cases of abdominal pain, which contributed to the marked decrease in the use of PT. In this case, endemic channels allowed us to interpret prescription habits according to a population phenomenon described not only at our clinic [15–17]. Historical comparison was important to detect these increases in DOT. Although major severity was considered the main cause of this PT increase, in January 2022, based on this finding, a new algorithm and educational strategies for intra-abdominal surgical infections were released at the institution to further optimize the use of PT.

*Vancomycin* This antibiotic is currently under strict surveillance, and we have noticed a progressive increase in the last year, especially related to severe methicillin-resistant *S. aureus* infections (prevalence > 42% at our center) and resistant pneumococcal infections. The increment identified this year was associated with severe cases of complicated pneumonia, without the identification of microbiological isolates and central nervous system infections. The considerable increase in PICU use compared to hospital use in general is related to the greater severity presented by this type of patient. More than 93% of prescriptions, according to ASP analysis, were considered adequate.

*Ampicillin-sulbactam* since January 2022, consumption has increased because of a change in the local recommendation (now is the first alternative in abdominal surgical prophylaxis), which explains this increase compared with previous years. This new recommendation implies that we need to update the EC according to new prescription habits and recommendations.

Recommendations about the implementation and interpretation of endemic channels from a Pediatric ASP:

- In constructing endemic channels, the use of 3 consecutive years of DOT is required to achieve the robustness needed for the mathematical method and the calculation of dispersion measures. Prefer the method of geometric means and confidence intervals over the method of median and quartiles, when computational capabilities allow it. Implementation of EC in critical services is fundamental.
- If any change in DOT to the alert zone is detected, it is recommended to implement strategies to increase the surveillance of that specific antibiotic and try to identify the origin of this variation (i.e. increase in cases of certain pathology, decline in local recommendation adherence, change in prescription habits, new personnel, and new recommendations) and, if considered appropriate, initiate educational strategies or ASP measures necessary to contain this increase in the consumption of antibiotics.
- Update the years of DOT used to construct endemic channels when modify local antimicrobial recommendations in the ASP for some antibiotics or when identify modifications in previous use because of epidemiological findings or changes (e.g., increase in MDR pathogens).
- Given the possibility of changes in local antibiotic use indications or clinical practice guidelines, it is recommended to reassess antibiotic consumption and the need to update endemic channel graphs every 2–3 years.
- Present endemic channels periodically to ASP committees and administrators, but also to clinicians to allow them to identify antimicrobial prescription habits across time.

## Conclusion

DOT is an important impact measure to monitor antibiotic consumption, and is useful for comparison with other hospitals and national standards when available. However, the use of endemic channels of prescription for surveillance of antibiotic use allows us to detect changes in prescriptions compared with historical use, and analyzing them will facilitate the understanding of when to turn on the alarms. Currently, at our center, EC is a useful tool to perform internal comparisons of antibiotic use and implement timely actions whenever needed. As part of our results and experience and considering the ease of its implementation, the use of EC methodology could be a helpful tool to implement in pediatric ASP.

## Data Availability

The datasets used and/or analyzed during the current study available from the corresponding author on reasonable request.
